# The Benefits of the Mediterranean Diet on Inflamm-Aging in Childhood Obesity

**DOI:** 10.3390/nu16091286

**Published:** 2024-04-25

**Authors:** Valeria Calcaterra, Elvira Verduci, Chiara Milanta, Marta Agostinelli, Federica Bona, Stefania Croce, Chiara Valsecchi, Maria Antonietta Avanzini, Gianvincenzo Zuccotti

**Affiliations:** 1 Pediatrics and Adolescentology Unit, Department of Internal Medicine, University of Pavia, 27100 Pavia, Italy; valeria.calcaterra@unipv.it; 2 Pediatric Department, Buzzi Children’s Hospital, 20154 Milan, Italy; chiara.milanta@unimi.it (C.M.); marta.agostinelli@unimi.it (M.A.); federica.bona@unimi.it (F.B.); gianvincenzo.zuccotti@unimi.it (G.Z.); 3 Department of Health Sciences, University of Milano, 20142 Milan, Italy; 4 Immunology and Transplantation Laboratory, Cell Factory, Pediatric Hematology Oncology, Fondazione IRCCS Policlinico S. Matteo, 27100 Pavia, Italy; stefania_croce186@yahoo.it (S.C.); chiara.valsecchi@unipv.it (C.V.); ma.avanzini@smatteo.pv.it (M.A.A.); 5 Department of Biomedical and Clinical Science, University of Milan, 20157 Milan, Italy

**Keywords:** Mediterranean diet, inflamm-aging, childhood obesity, children, nutrition, pediatric

## Abstract

(1) Background: Numerous elements of the Mediterranean diet (MD) have antioxidant and anti-inflammatory qualities. (2) Methods: We present a narrative review of the potential benefits of the Mediterranean dietary pattern (MD) in mitigating aging-related inflammation (inflamm-aging) associated with childhood obesity. The mechanisms underlying chronic inflammation in obesity are also discussed. A total of 130 papers were included after screening abstracts and full texts. (3) Results: A complex interplay between obesity, chronic inflammation, and related comorbidities is documented. The MD emerges as a promising dietary pattern for mitigating inflammation. Studies suggest that the MD may contribute to weight control, improved lipid profiles, insulin sensitivity, and endothelial function, thereby reducing the risk of metabolic syndrome in children and adolescents with obesity. (4) Conclusions: While evidence supporting the anti-inflammatory effects of the MD in pediatric obesity is still evolving, the existing literature underscores its potential as a preventive and therapeutic strategy. However, MD adherence remains low among children and adolescents, necessitating targeted interventions to promote healthier dietary habits. Future high-quality intervention studies are necessary to elucidate the specific impact of the MD on inflammation in diverse pediatric populations with obesity and associated comorbidities.

## 1. Introduction

Aging is characterized by a gradual and continuous progression of natural changes across biological, physiological, immunological, environmental, psychological, behavioral, and social processes [1]. Inflamm-aging (IA) is characterized by chronic, low-grade inflammation that exacerbates the aging process and related chronic diseases. Immune dysregulation, starting from childhood [2], is a crucial factor in the development of chronic inflammation [1]. A number of environmental insults, lifestyles, and nutritional factors may play an important role in immuno-modulation and IA in non-communicable chronic disease (NCD) pathogenesis, including obesity and related metabolic risk. Specifically, children with obesity are individuals with a high cumulative biological risk. In this risk group, cellular senescence can occur due to various types of cellular stress and is characterized by a pro-inflammatory secretory phenotype, potentially leading to premature aging mechanisms [2].

In 2019, about 38.2 million children under the age of 5 worldwide were overweight or obese [3], and it seems the SarsCov2 pandemic exacerbated the situation. A recent study analyzed the electronic health records from March 2019 to January 2021 of 191,509 American citizens aged 5 to 17; it found an increase in overweight and obesity, especially among children aged 5 to 11, where the rate rose by 8.7% during the pandemic. Currently, 45.7% of children aged 5 to 11 are overweight. Compared to pre-pandemic levels, overweight rates in teenagers aged 12 to 15 increased by 5.2% [4]. This rise in childhood obesity has contributed to the global increase in chronic diseases associated with excess weight, including adult obesity, cardiovascular disease, high blood pressure/hypertension, renal comorbidities, gastrointestinal diseases like non-alcoholic fatty liver disease (NAFLD), non-alcoholic steatohepatitis (NASH), cholelithiasis, hepatocellular carcinoma, and pulmonary diseases such as asthma and obstructive sleep apnea [5].

An important link between obesity and its comorbidities is the pro-inflammatory nature of obesity [6]. Indeed, obesity is associated with chronic low-grade inflammation, stemming from metabolic issues resulting from cells’ heightened exposure to fatty acids [7]. When adipose tissue’s storage capacity is surpassed, free fatty acids accumulate in ectopic locations, potentially leading to cellular dysfunction, cell death, and inflammation [8]. This leads to the increased secretion of hepcidin by the liver, a small peptide hormone that serves as a homeostatic regulator of systemic iron metabolism and also acts as a mediator of host defense and inflammation [9]. Furthermore, inflammation is characterized by alterations in the levels of cytokines and acute-phase reactants in the blood. The inflammatory cascade is primarily executed by M1 macrophages and the Toll-like receptor family, notably Toll-like-receptor 4 (TLR4). TLR4 binds to the ligands’ lipopolysaccharides (LPSs), initiating a signaling pathway that results in the production of nuclear factor κB (NFκB) and cytokines, predominantly IL-6, IL-1, and TNF-α, as well as serum amyloid A3, alpha 1-acid glycoprotein, lipocalin 24p3, and plasminogen activator inhibitor-1 (PAI-1). The Mediterranean diet (MD) is recognized as an effective diet for maintaining a healthy weight and preventing obesity. It is characterized by a high intake of vegetables, fruits, whole-grain products, nuts, seeds, olive oil as the primary fat source, a moderate consumption of dairy products daily, and some servings of poultry or fish weekly [10]. Moreover, numerous elements of the MD have antioxidant and anti-inflammatory qualities. Among them, there are biologically active nutrients, including polyphenols, which are important for the management and prevention of chronic non-communicable illnesses thanks to their advantageous anti-oxidative and anti-inflammatory properties [11]. Few studies have revealed a statistically significant difference in body mass index (BMI) or other adiposity measures between the diet intervention and the control group. However, an MD can support the maintenance of healthy body weight and prevent obesity through a variety of plausible mechanisms [12].

In this review, we revised the mechanisms underlying chronic inflammation in obesity and the benefits of Mediterranean dietary patterns on IA in childhood obesity, focusing on the effectiveness of the diet in the treatment of excess weight and related complications. Even though IA is usually described in elderly subjects, this concept may be extended to pediatrics with respect to subjects with high cumulative biological risk, such as obesity and/or other chronic conditions [2]. The MD can not only be supported and proposed as an effective dietary pattern for treatment but can also prevent inflamm-aging and related comorbidities from early life stages and onwards. The sharing of opinions provides the power to create opportunities for debates on the topic and can reflect in the strategies for managing obesity and obesity-related complications using a nutritional approach.

## 2. Methods

**Figure 1:**
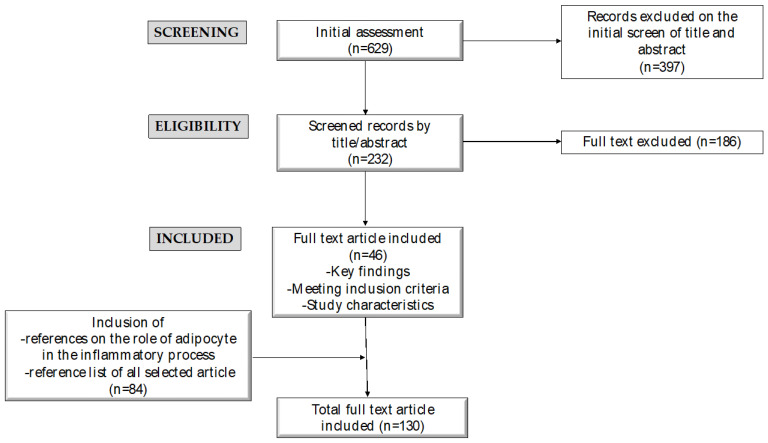
Manuscript selection and exclusion process used in writing this narrative review.

We performed a narrative review to revise the mechanisms driving low-grade inflammation in obesity and investigate the impact of the Mediterranean dietary pattern on inflamm-aging in pediatric populations with obesity, with a specific focus on the positive effects of weight control and comorbidities associated with inflammation. The main focus of the research was children and adolescents; studies involving adults were also included to establish the context and highlight the potential impact of MD. We conducted an extensive literature search on PubMed, with the language being restricted to English. The most pertinent original scientific papers, clinical trials, meta-analyses, and reviews published over the past 15 years were taken into account. We incorporated original research articles, reviews, meta-analyses, and clinical practice guidelines. Case reports and case series were excluded due to their typically low level of evidence. The research terms adopted were “childhood obesity” and/or “Mediterranean diet” and/or “Chronic inflammation”. The initial search retrieved 629 records, and we assessed 232 abstracts; we excluded 186 studies based on their abstracts, and 46 full texts were evaluated. Furthermore, references concerning the role of adipocyte dysfunction in the inflammatory process associated with obesity and the reference lists of all articles were examined to identify relevant studies (n = 84), and ultimately, 130 papers were included. Figure 1 illustrates the paper selection and exclusion process.

## 3. Relationship between Aging, Low-Grade Inflammation, and Pediatric Obesity-Related Complications

### 3.1. Aging and Chronic Low-Grade Inflammation 

Aging is a physiological process that occurs in all organisms, and it is characterized by a gradual decline in molecular and biological functions. At this stage, cells and tissues respond less efficiently to stress and damage, exhibiting increased vulnerability relative to death and disease. The resulting oxidative damage and metabolic imbalances, together with immunological impairment, essentially cause a reduced capacity to manage persistent types of inflammation. The molecular basis of inflammation has been described, and different molecules are reported to be linked to related chronic diseases [13,14]. Pro-inflammatory (i.e., tumor necrosis factor (TNF)-α, interleukin (IL)-1, IL-6, interferon (IFN)-α, transforming growth factor (TGF)-β) and anti-inflammatory cytokines (IL-1 receptor antagonists: IL-4, IL-10, IL-13, and IL-33) are important components of the immune system, and their age-associated dysregulation may adversely affect the immune response. While the release of cytokines in acute inflammation induces a systemic response, resulting in the destruction of pathogens and tissue repair, in chronic inflammation, the persistent signal disturbs the restoration of damaged tissues.

Chronic low-level inflammation, associated with the aging process, has been reported to be a risk factor for several chronic diseases [15].

The possible causes of chronic inflammation have been ascribed to genetic susceptibility, chronic infections, changes in gut microbiota and permeability, and obesity [16].

Genetic susceptibility is responsible for inter-individual variations through a network of factors.

Single-nucleotide polymorphisms (SNPs) have been described to be associated with the development of age-related diseases. For example, SNPs in the C-reactive protein (CRP) gene increase the risk of myocardial infarction in cardiovascular disease (CVD) [17]. In contrast, an SNP in the promoter region of IL-6 (174G > C), enhancing IL-6 production in response to inflammatory stimuli, increases the risk of Alzheimer’s disease, non-insulin-dependent diabetes mellitus, and juvenile chronic arthritis [18,19,20].

There is evidence that suggests that epigenetic changes, such as DNA methylation and histone modifications, are connected with aging, resulting in the overexpression of pro-inflammatory genes. On the other hand, a persistent inflammatory state leads to the accumulation of DNA damage [21].

Some cellular changes have been described to be mediated by microRNAs (miRNAs), which are non-coding, single-stranded RNAs that are involved in gene expression modulation via the reduction of mRNA stability or mRNA translation [22]. An association between miRNAs and age-related pathologies was reported, and low levels of miR-126–3p were found in patients with cardiovascular disease (CVD) and diabetes, while miR-21–5p levels were higher in patients with CVD than in age-matched controls [23,24,25,26,27].

Chronic infections—for example, those due to cytomegalovirus (CMV), hepatitis C virus (HCV), or human immunodeficiency virus (HIV)—also play a role in IA. In these pathological conditions, the immune system is continuously stimulated and induced to produce high levels of pro-inflammatory cytokines that cause a chronic inflammation state [28,29].

In recent years, increasing evidence has demonstrated the linkage between low-grade chronic inflammation and intestinal dysbiosis: the intestinal flora continuously stimulates the immune system in order to ensure rapid and effective defense against pathogens [30,31,32].

The composition of gut microbiota is heterogeneous throughout the intestinal tract and is particularly sensitive to external insults. The intestinal homeostasis represented by the balance of different bacterial species is indeed fundamental [31,33]. However, age-related changes in human intestinal microbiota are physiological and depend on individual characteristics and lifestyles [34,35,36].

Intestinal dysbiosis, demonstrated in elderly people, has been described as a cause of local inflammation and T-cell activation in the systemic compartment [37,38,39,40,41,42,43].

Moreover, lifestyle and environmental factors, such as obesity, radiation, alcohol, tobacco, or toxicants, are to be taken into account [44,45].

Currently, obesity is considered a pathological condition that is strongly associated with a variety of age-related inflammatory diseases, including cardio-metabolic disorders and cancer [46,47].

In obesity, adipose tissue is characterized by both the increase in adipocyte size (hypertrophy) and number (hyperplasia). Adipocyte hypertrophy results in the reduced uptake and storage of fatty acids and an increase in lipolysis, inflammatory cell infiltration, and adipokine secretion, determining the so-called “lipotoxicity” [45]. Under lipotoxic conditions, these fatty acids are stored as lipid cells, or fat, in ectopic tissues, such as the liver, pancreas, heart, and skeletal muscle, and they are not designed to support excessive fat [46]. Intracellular accumulation and the cell’s inability to use fatty acids lead to cellular dysfunction, with an increase in reactive oxygen species (ROS) production and alterations in lipidic cellular membrane composition [48].

Chronic low-grade inflammation, often observed in patients with obesity, could be ascribed to adipocyte organelle dysfunction or adipose tissue hypoxia. An excess of energy causes mitochondria malfunction, with an increase in ROS production. The subsequently induced redox-sensitive transcription factors, such as NF-κB, stimulate adipokine secretion [48]. On the other hand, the “hypoxia theory” renders localized hypoxia the primary catalyst for adipokine dysregulation in obesity [49].

Additionally, adipokines—including adiponectin, leptin, resistin, visfatin, apelin, vaspin, hepcidine, IL-1, IL-6, IL-8, IL-10, transforming growth factor β-1 (TGF-β1), TNF-α, chemerin, omentin, monocyte chemoattractant protein-1 (MCP-1), retinol-binding protein-4, and plasminogen activator protein (PAI)—have been described to cover different roles in metabolic and inflammatory responses that change with respect to the site of fat depots. All these observations render the AT an endocrine organ, and its functional dysregulation leads to low-grade chronic inflammation diseases [50].

In obesity, the infiltration of macrophages, monocytes, and T and B lymphocytes is observed in the adipose tissue, and the accumulation of immune cells is correlated with the body mass index [51]. In particular, an increase in T lymphocytes that secrete IFN-α and TNF-α, which are related to insulin resistance, has been documented [52,53].

Despite all reported aspects, more efforts must be carried out to understand the alterations caused by obesity in order to better treat this pathological condition, particularly in pediatric settings.

### 3.2. Inflammation and Its Role in Complications Related to Pediatric Obesity

Little evidence suggests that the inflammation state is implicated in clinically important childhood obesity complications, including IR, diabetes, cardiovascular, respiratory and gastrointestinal disorders, and NAFLD [54]. Low-grade chronic inflammation can already be observed in preschool children [55].

As previously described in detail, the enlarging adipose tissues in subjects with obesity synthesize and secrete hormones and proteins such as leptin, adiponectin, TNF-α, and other cytokines, which modify insulin secretion and sensitivity, resulting in insulin resistance [8] and increasing the risk of developing T2D.

A significant connection between autoimmune illnesses and low-grade chronic inflammation has been described; a significant study found that children with obesity had a higher prevalence and worse prognosis for a number of autoimmune disorders, including psoriasis, inflammatory bowel disease, and systemic lupus erythematosus [56].

Obesity and correlated chronic low-grade inflammation are also strictly associated with increased cardiovascular risk, and this is mainly due to hypercholesterolemia, hypertension, endothelial dysfunction, and/or non-nocturnal dipping blood pressure [55].

Additionally, inflammation can contribute to NAFLD, which is currently the most prevalent liver disease in children with obesity [57]. The liver plays a crucial role in glucose regulation, and liver dysfunction, coupled with inflammatory factors, heightens the risk of diabetes [58]. However, the relationship is bidirectional, as diabetes is also linked to impaired liver function and an increased likelihood of NAFLD progressing to fibrosis and non-alcoholic steatohepatitis [59].

Childhood obesity also affects bone health. Although bone mass is positively associated with body weight, it has been reported that it may be negatively influenced by endocrine and/or paracrine factors that are associated with obesity and affect bone synthesis and vitamin D levels [60].

The hormonal and pro-inflammatory effects of adipose tissue have also been suggested to account for the observed association between obesity and asthma [61]. Considering the epidemiological link between the two diseases and the common feature of the abnormal activation of inflammation pathways, it is reasonable to hypothesize a common mechanism of action, although the exact mechanisms are still unknown [62].

To summarize, evidence in the literature strongly suggests that chronic low-grade inflammation has a key role in obesity pathogenesis and its major complications. However, more studies are needed to expand on the association between the two conditions.

**Figure 2:**
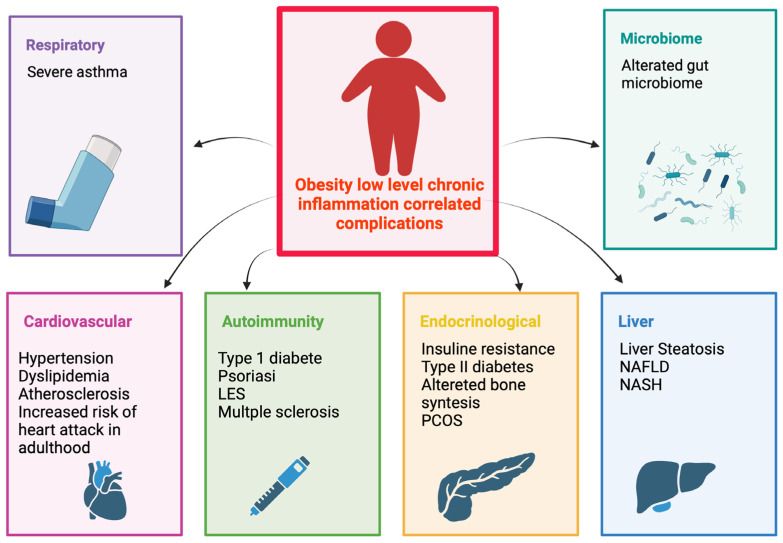
Chronic low-grade inflammation-related complications. Created with BioRender.com. Abbreviations: non-alcoholic steatohepatitis (NASH); systemic lupus erythematosus (LES).

The main chronic low-grade inflammation types discussed are summarized in Figure 2.

## 4. Mediterranean Diet (MD) and Inflamm-Aging

### 4.1. Anti-Inflammatory Properties of the Mediterranean Diet (MD)

The MD is an environmentally sustainable dietary pattern characterized by a high intake of plant-based foods, including vegetables, fruits, whole-grain cereals, legumes, nuts, and seeds. It also emphasizes a moderate-to-high consumption of fish and seafood, a moderate intake of eggs, poultry, and dairy products (milk, yogurt, and cheese), and limited consumption of red meat. Olive oil, abundant in unsaturated *n*-9 fatty acids, serves as the primary source of added fat [63]. Both animal and human studies have elucidated the biological mechanisms underlying the beneficial effects of the traditional MD, including lipid-lowering, anticancer, antimicrobial, and anti-inflammatory properties [64].

The interaction between nutrition and the immune system is highly intricate. Specifically, at each phase of the immune response, certain micronutrients play pivotal and often synergistic roles. Deficiency in even a single essential nutrient can compromise the immune system [65]. The possible mechanism behind the pro-health effect of the MD could be due to its immunomodulatory and anti-inflammatory properties [66,67,68,69]. The MD is rich in components such as monounsaturated fatty acids (MUFAs), omega-3 fatty acids, polyphenols, flavonoids, phytosterols, vitamins (b-carotene, vitamin C, and vitamin E), and minerals with antioxidant and anti-inflammatory activity (such as selenium and micronutrients).

A high vegetable and fruit intake is associated with a lower hs-CRP [70,71] and IL-6 [72]. Moreover, data from the 1999 to 2002 National Health and Nutrition Examination Survey (NHANES)‘s cross-sectional study show that children and adolescents with higher levels of CRP had significantly lower intakes of grains and vegetables [73].

Extra virgin olive oil, a cornerstone of the MD, is abundant in antioxidant, anti-inflammatory, and immune-modulating compounds. These are primarily monounsaturated fatty acids, especially oleic acid, and constituents of the unsaponifiable fraction, comprising about 2% of the oil’s weight. This fraction includes polyphenols, phytosterols, tocopherols, and pigments [74]. Among these compounds, polyphenols seem to mitigate inflammation by functioning as antioxidants, inhibiting the production of pro-inflammatory cytokines; suppressing inflammatory diseases; inducing metabolic gene expression; or activating transcription factors that counteract chronic inflammation [75] In addition, oleocanthal, a polyphenol found in olive oil, contributes to inhibiting the activity of cyclooxygenases 1 and 2 (COX1 and 2), which are key enzymes of the inflammatory process, catalyzing the synthesis of prostaglandins and suppressing the lipopolysaccharide-mediated upregulation of pro-inflammatory factors, such as IL-1, IL-6, and TNF-a [76]. Moreover, wine, especially red wine, has specific polyphenols with antioxidant properties, including resveratrol, procyanidins, and monophenols [77].

The current mechanisms that underpin the beneficial effects of the Mediterranean diet (MD) encompass enhanced lipid profiles, insulin sensitivity, and endothelial function, along with anti-thrombotic properties [78]. These effects are most likely due to bioactive ingredients like polyphenols, mono- and polyunsaturated fatty acids—particularly oleic acid found in olive oil—and dietary fibers [79].

Omega-3 polyunsaturated fatty acids (PUFAs), which are prevalent in the Mediterranean diet (MD), exhibit immunomodulatory effects. They influence leukocyte chemotaxis, adhesion molecule expression, and leukocyte-endothelium adhesive interactions. They also affect the production of eicosanoids like prostaglandins and leukotrienes from arachidonic acid and promote the production of anti-inflammatory cytokines. Specifically, they diminish the expression of pro-inflammatory factors such as IL-1, IL-6, TNF, VCAM-1, and MCP-1; reduce levels of reactive oxygen species (ROS) and nitrogen species; and simultaneously elevate anti-inflammatory cytokines like IL-10 [80,81]. Moreover, they modulate T-cell function both directly, by inhibiting the differentiation of Th1 and Th17 cells, and indirectly, by impeding the function of antigen-presenting cells like monocytes/macrophages and dendritic cells [82].

Sured et al. found that greater adherence to the MD among adolescent girls was associated with lower levels of C-reactive protein (CRP) and leptin [83]. A cross-sectional analysis involving 1462 adolescents (aged 9–18) yielded similar results [84]. Additionally, the MD has been reported to reduce levels of IL-1, IL-2, IL-6, and TNF-α [85]. Lastly, in a study involving 44 children with asthma following the MD, a reduction in IL-17 levels was observed [86].

In addition, other research studies conducted in the adult population suggest that the long-term consumption of an MD may be an effective strategy for protection against metabolic syndrome, a risk factor for type 2 diabetes mellitus, and cardiovascular diseases [87,88].

Bioactive compounds in the MD also seem to play an epigenetic role by modifying molecular parameters like methylation profiles and microRNA expression, which are linked to inflammation modulation [89,90]. Specifically, polyphenolic compounds have been linked to DNA methylation changes in various cancer-related genes, as well as in key tumor suppressors and promoters [91]. Anthocyanins, pigments found in berries, eggplants, black grapes, pomegranates, and cruciferous vegetables, have demonstrated an ability to influence the cell cycle in vitro through epigenetic modifications, thereby stimulating DNA repair mechanisms [92].

Fisetin, a flavonoid found in apples, cucumbers, strawberries, onions, and persimmons, has been shown to inhibit cancer cell growth, leading to changes in various signaling pathways, including cell division, angiogenesis, metastasis, oxidative stress, and inflammation [93]. Quercetin, another flavonoid present in berries, cruciferous vegetables, red grapes, red onions, tomatoes, and citrus fruits, has been associated with the inhibition of tyrosine kinase Janus kinase 2, which is known to induce apoptosis and autophagy in cancer cells [94]. In Table 1, evidence from the literature on the bioactive compounds present in the foods characteristic of the MD is shown.

Recent studies have shown that the consumption of an MD is strongly associated with a reduction in subclinical intestinal inflammation through the modulation of the gut microbiota [95]. These findings are consistent with studies in humans, where increased levels of SCFAs have also been described. In particular, the abundance of *Enterorhabdus*, *Lachnoclostridium*, Prevotella *Parabacteroides*, and fiber-degrading *firmicutes,* as well as lower *Escherichia coli* counts, has been reported [96]. A high fiber intake also promotes the release of metabolites, such as short-chain fatty acids (e.g., acetate, propionate, butyrate), which regulate immune functions, as mentioned previously [97].

**Table 1:** Literature evidence on the bioactive compounds present in the food characteristic of the Mediterranean diet.

Sources	Foods	Metabolic Properties	References
PUFAs Omega-6 linoleic acid	Vegetable oil (grapeseed, wheatgerm, soya, corn, sunflower seed, sesame, rice, canola, peanut, almond); frying oil, walnuts, margarine, cod liver oil, and pork fat)	Positive modulation of leukocyte and T- cell function ↑ Anti-inflammatory cytokines (IL-10) ↓ Pro-inflammatory cytokines (i.e., IL-6, IL-1, TNF-a, NF-kB) ↓ ROS, nitrogen species, platelet-activating factor (PAF), adhesion molecules (ICAM-1, VCAM-1, and selectins) and chemokines (IL-8 and MCP-1)	[80,97,98,99]
Omega-6 arachidonic acid	Beef, bone marrow, lard, chicken, and cod liver oil		
Omega-3 alpha-linolenic acid (ALA)	Linseed, vegetable oil (canola, rapeseed, soya, wheatgerm, and palm), walnut, and seeds		
EPA and DHA	Cod liver oil, mullet, salmon, mackerel, tuna, grouper, anchovies, and sardines		
MUFAs	Vegetable oil (olive, almond, canola, rapeseed, macadamia, peanut, pecan nut, sesame, rice, and palm), cod liver oil, lard, beef tallow, pistachio nuts, and margarine	↓ Proinflammatory cytokines (i.e., IL-6, IL-1, TNF-a)	[98,100]
Polyphenols: phenolic acids, flavonoids (fisetin and quercetin), stilbenes, phenolic alcohols, and lignans	Fruits (grapes, berries, apples, cucumbers, strawberries, persimmons, and citrus fruit), vegetables (onions and tomatoes), cereals, olives, dry legumes, chocolate, beverage (tea, coffee, and red wine), and some spices	↓ Proinflammatory cytokines Antioxidant properties ↑ Transcription of anti-inflammatory mediator ↓ COX1-2 ↓ LPS → ↓ IL-6, IL-1, and TNF-a ↑ Lipid profile ↑ Insulin sensitivity ↑ Endothelial function Modulation of gene expression	[75,76,101]
Vitamins	Vitamin C: grape, guava, peppers, orange, blackcurrant, nettle, parsley, lemon, tomatoes, kiwi, broccoli, and apricot ß-carotene: paprika, parsley, tomato, seaweed, apricot, carrot, basil, peppers, marjoram, mint, valerian, and vegetable oil	Antioxidant properties Anti-inflammatory action	[98,102,103]
	Vitamin E: vegetable oil (wheatgerm, corn, sunflower seed, almond, palm, rice, and mixed seeds)	Modulate gene expression Protects cell membranes and supports the integrity of epithelial barriers ↓ PGE2; ↑ IL-2; ↑ NK cell cytotoxic activity	[65,98,104]
	Vitamin B 12: beef, horse, sheep’ liver, lamb, and clams Vitamin B 6: wheat germ, corn and olive oil, yeast, pistachio nuts, and tempeh Folate: leavening agents, yeast, baker’s, active dry, chicken, and liver	↑ NK cell cytotoxic activity Modulates lymphocyte proliferation, differentiation, maturation, and activity	[97,98]
	Vitamin D: cod liver oil, herring, salmon, catfish, and egg yolk	↑ Integrity of mucosal cells in innate barriers Regulates antimicrobial proteins (cathelicidin and b-defensin) ↓ IFNc and IL2; ↓ T-cell proliferation ↓ antibody production by B cells ↑ monocytes differentiation to macrophages; ↑ movement and phagocytic ability of macrophages	[98,105]
Minerals (Ca, P, K, Fe, Mn, Zn, Selenium)	Calcium: basil, marjoram, thyme, roe, oregano, mint, milk, cow, shimmed, rosemary, cinnamon, and grana cheese Phosphorus: yeast, wheat bran, sea bass, wheat germ, gilthead bream, milk, eggs, and chicken Iron: liver, beef, veal, spleen, pork, poultry, fish, and legumes Magnesium: wheat bran, cocoa, coffee, grey mullet, roe, caviar, pine nuts, and almonds Potassium: mushrooms, leavening agents, seaweed, soya, tea, and flour	Anti-inflammatory and antioxidant actions	[98,106]
	Zinc: mollusks, oyster, eastern, canned cheese made with cow milk, agar, and mushrooms	Maintains or enhances NK cell cytotoxic activity Helps modulate cytokine release by dampening the development of pro-inflammatory Th1 cells and influencing the NK cell generation and cytokines (i.e., IL-2, IL-6, TNF-a)	[97,98,106,107]
	Selenium: nuts, cod, beef, kidney, and tuna	Cellular antioxidants (↓ ROS produced during oxidative stress)	[65,98,106]
Soluble fiber	Figs, carrots, kiwifruit, nectarines, peaches, pears, melons, oranges, lettuce, and broccoli	↑ GUT microbiota → ↑ SCFAs (acetate, propionate, and butyrate)	[65,106]
Insoluble fiber	Figs, pears, broccoli, kiwifruit, carrots, oranges, and lettuce		

Abbreviation: LPS (lipopolysaccharide); IL (interleukin); COX 1-2 (cyclooxygenases 1 and 2); MUFA (monounsaturated fatty acids); PUFA (polyunsaturated fatty acids); NK (natural killer); Th (T helper cells); TNF (tumor necrosis factor); ROS (reactive oxygen species); NF-kB (nuclear factor kappa-light-chain enhancer of activated B cells); PGE2 (prostaglandin E2); ALA (omega-3 linolenic acid); EPA (eicosapentaenoic acid); DHA (docosahexaenoic acid); ↓ decrease; ↑ increase.

**Figure 3:**
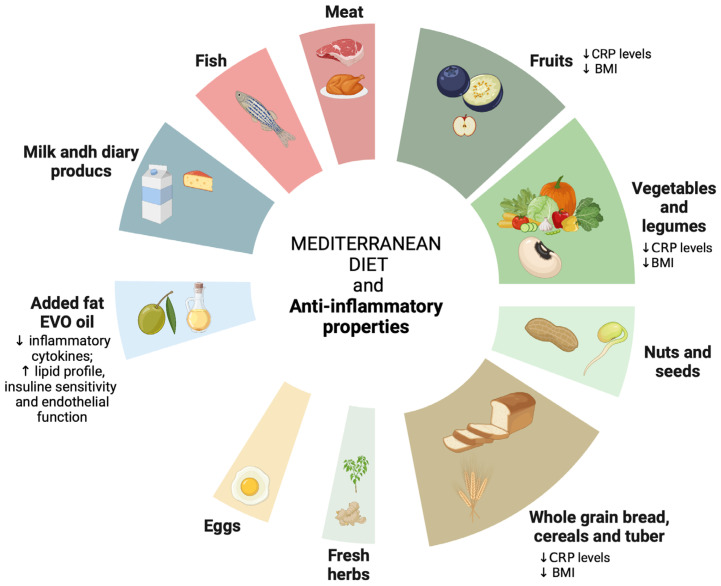
Anti-inflammatory properties of MD are schematized. Created with BioRender.com. ↓ decrease; ↑ increase.

More studies in pediatric populations are needed to define the anti-inflammatory role of the MD in even greater detail. In Figure 3, the anti-inflammatory effects of MD are schematized.

### 4.2. The Effectiveness of Mediterranean Diet Adherence in Children with Obesity

The MD has numerous beneficial effects, particularly for adults with cardiovascular diseases. However, there are limited data on its effects in reducing the risk of obesity, insulin resistance (IR), and metabolic syndrome (MetS) in children and adolescents [108]. Research has demonstrated that adhering to the MD is crucial for preventing childhood obesity and maintaining a healthy body weight [109]. This diet emphasizes the consumption of high volumes of low-energy foods like fruits and vegetables, which take longer to digest, potentially increasing satiety and reducing overall energy intake. Moreover, the MD promotes the intake of high-fiber and nutrient-rich foods, resulting in reduced calorie consumption [110]. Additionally, the MD includes components such as phenolic compounds in olive oil, omega-3 polyunsaturated fatty acids, vitamins, and trace elements, which are important for shaping the composition of gut microbiota. On the other hand, gut dysbiosis is linked to an increased risk of obesity and metabolic syndrome [109,111]. In contrast, the consumption of ultra-processed foods is associated with a higher risk of noncommunicable diseases [112]. Numerous studies have shown that ultra-processed foods typically have a higher energy content, more free sugars and unhealthy fats, and less fiber, protein, and micronutrients compared to minimally processed foods. Consumption of these products has been linked to poorer nutritional status [113,114].

One of the main problems of our time is that the traditional MD is mainly followed by the elderly population, while a very low adherence in the pediatric population has been reported [115,116].

Scientific evidence suggests that the MD has inverse associations with obesity and MetS indicators, such as high BMI [117], waist circumference (WC) [118], insulin resistance, and high lipid levels [119]. Furthermore, it has been reported that following this diet reduces the risk of chronic diseases [119,120]. Many studies have also reported a significant reduction in mortality rate [121]. In contrast, low MD adherence is associated with high risks of central obesity, hypertriglyceridemia, and insulin resistance.

Velasquez-Lopez et al. [119] reported that MD is linked to a decreased BMI; reduced fat mass; and lower blood glucose, total cholesterol, triglyceride, HDL-C, and LDL-C levels. Following this diet also increased the consumption of omega-9 fatty acids and various micronutrients, such as zinc, vitamin E, and selenium, and it decreased the consumption of saturated fatty acids, which are associated with a worse nutritional status.

A Greek study [122] investigated the link between adherence to the Mediterranean diet (MD) and childhood overweight/obesity, considering family structure as a contributing factor. The study found that greater adherence to the MD acts as a protective factor against childhood overweight/obesity, particularly among children living with their biological families. Family structure appears to be a significant determinant of weight status in children. The authors concluded that following the MD could reduce the prevalence of pediatric overweight/obesity.

Barandianan et al. [117] carried out both cross-sectional and longitudinal studies to evaluate the risk of obesity in children. They discovered that there was no association between adherence to the Mediterranean diet (MD) and overweight, obesity, or abdominal obesity at the age of 4. However, they did find that high adherence to the MD at the age of 4 was linked to a reduced risk of overweight, obesity, and abdominal obesity by the age of 8.

Bacopoulou et al. [118] observed a group of adolescents aged 12–17 who underwent dietary assessments using the MD Quality Index (KIDMED), blood pressure (BP), and obesity assessments both at baseline and after a 6-month school-based healthy diet. They reported a significant decrease in overweight and obesity, mean systolic and diastolic BP, WC (waist circumference), and WHtR (waist/height ratio). They also reported a decrease in WC as the KIDMED score increased. The authors suggest that multilevel school-based interventions may help reduce and prevent adolescent overweight and obesity.

De Santi et al. [123] conducted a cross-sectional study to assess adherence to the Mediterranean diet (MD) and its association with weight status among a group of Italian middle school adolescents, using the KIDMED test. The study found that 26.8% of the adolescents were overweight, and 11.7% were obese. Adherence to the MD was high in 13.3% of the students, average in 27.1%, and low in 59.6%. No significant differences were observed in terms of gender and age. The authors concluded that there is very low adherence to the MD among adolescents living in Mediterranean countries, highlighting the need to promote the importance of the MD in reducing childhood obesity.

Seral-Cortez et al. [124] released a narrative review in which they analyzed all studies regarding gene–MD interaction effects and their associations with changes in body composition. They found that high adherence to the MD in individuals with a limited number of risk alleles was associated with a lower risk of adiposity and MetS. Moreover, they observed sex-specific differences, with a higher risk in genetically predisposed females than in males. These results were reported in the HELENA study, a cross-sectional multicentric study of European adolescents [125]. No other studies regarding gene–MD interaction in children were found.

Lopez-Gil et al. [109] made a systematic review to evaluate the effects of MD-based interventions on anthropometric and obesity indicators in children and adolescents. They analyzed 15 randomized controlled trials (RCTs). It was observed that the MD-based lifestyle is linked to a small but significant reduction in BMI and the percentage of obesity. However, small and nonsignificant decreases in WC, WHtR, and the percentage of abdominal obesity were observed.

Previous systematic reviews have yielded inconclusive findings [126,127], primarily due to the limited number of randomized controlled trials included in the meta-analyses. Iaccarino Idelson et al. [126] reported mixed and contrasting results; out of 26 papers, only 10 indicated that higher adherence to the MD was associated with lower BMI values or a reduced prevalence of overweight. The authors predominantly analyzed observational studies, which could account for these inconsistent findings. Lassale et al. [127] concluded that the only discernible benefit of the MD lies in maintaining a healthy body weight during childhood. Their research was solely based on the MEDLINE database, overlooking several significant studies.

Emerging scientific evidence suggests that the MD may be effective in preventing metabolic syndrome in children and adolescents with obesity. Yurtdaş et al. [128] conducted a study to evaluate the efficacy of the MD in reducing metabolic syndrome indicators in children and adolescents with obesity. They found that following an MD for 12 weeks led to reductions in BMI, fat mass, hepatic steatosis, and insulin resistance; improved transaminase levels; and had positive effects on inflammation and oxidative stress in adolescents with obesity and non-alcoholic fatty liver disease (NAFLD). However, the impact of the MD on pediatric populations remains insufficiently explored, warranting further studies to assess its effectiveness in mitigating cardiovascular risk factors and non-communicable diseases.

Literature evidence on the MD effects and adherence in children and adolescents with obesity is reported in Table 2.

## 5. Limitations 

As Gregory et al. [129] pointed out, a narrative review provides a non-systematic overview and analysis of the existing literature on a particular topic. Due to its non-systematic nature, there are no formally established guidelines for conducting narrative reviews, which can lead to potential biases in selection and often results in qualitative syntheses.

Therefore, our review methodology has inherent limitations. Specifically, our article search was confined to publications from the last 15 years, and only articles from PubMed were included. While the primary focus of the research was on children and adolescents, the manuscript also incorporated studies that involve adults to set the context and emphasize the potential impact of the MD. In our selection criteria, we decided to exclude case reports and case series because they typically exhibit low levels of evidence. However, it cannot be ruled out that some of these reports may include descriptions supporting the topic of the review.

Furthermore, the scarcity of observational studies on pediatric patients underscores the need for additional research to establish robust evidence regarding the benefits of the MD on inflamm-aging in childhood obesity.

## 6. Conclusions

Most studies indicate that the MD plays a significant role in lowering the risk of obesity in children and adolescents. Children should be offered a well-balanced diet to minimize the risk of developing chronic diseases. The benefit of the MD is largely due to its anti-inflammatory effect, as discussed above. The MD has a strict link with the immune response due to its immunomodulatory and anti-inflammatory properties. Conversely, the concept of IA is relatively new, and in pediatrics, it remains poorly established. However, as outlined in the literature, this concept can also be applied to pediatrics, especially in relation to individuals with high cumulative biological risk factors, such as obesity.

Overall, the dietary patterns based on the principles of the MD are recognized by several international societies as promising approaches to obesity prevention in those of a pediatric age; however, this recommendation is mainly based on expert opinion, as there is a lack of evidence. Studies examining the impact of the Mediterranean dietary pattern on IA in childhood obesity have recently emerged, and they are promising. The dissemination of the principles of the MD should be consistently supported, focusing on its beneficial effects.

Unfortunately, despite the MD benefits, in Mediterranean countries, adherence to it among children and adolescents is very poor. Dietary habits are shifting toward a “Western diet” that is richer in saturated fat, simple carbohydrates, ultra-processed foods, and junk foods [130]. Scientific evidence suggests that Mediterranean populations are changing their traditional eating habits [115,131]. Moreover, traditional food choices in Italy, Greece, and other Mediterranean regions are being abandoned, especially among children; this trend could be caused by the globalization of the food supply [130].

Overall, the nutritional principles of the MD can be transcultural if an appropriate adaptation is provided using culture-specific foods. This might be a promising strategy for increasing the compliance of children and adolescents. Educating parents and caregivers while adopting innovative educational approaches is crucial in order to raise awareness about the importance of a balanced diet and the maintenance of healthy body weight, particularly in low socioeconomic areas. Moreover, since it appears that healthy habits are usually and more easily learned in the first years of life, although they are not directly associated with adherence to the MD [115], it is important that children attending full-time school have the opportunity to eat in a proper cafeteria with a variety of healthy options. In doing so, they would be encouraged to taste new flavors, following the examples set by their peers. This aspect would be even more important for children who do not receive proper nutritional education at home.

**Figure 4:**
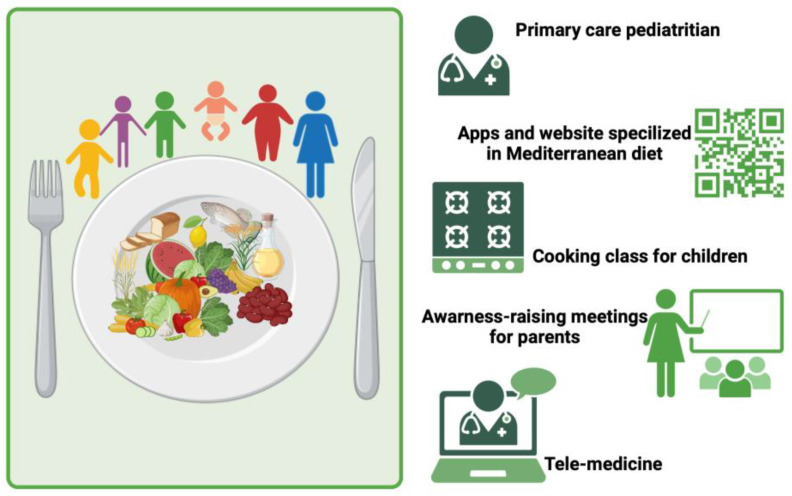
Possible actions to spread the Mediterranean diet. Created with BioRender.com.

Furthermore, the dissemination of MD principles can be encouraged through specialized and scientific-based websites or applications. These tools might promote adherence to the MD by providing examples of healthy dietary patterns and daily menus for entire families. These platforms could also provide users with shopping lists, encouraging them to purchase fresh and healthy foods at the supermarket. Children should have the opportunity to attend cooking classes, where they can have fun while experimenting with new flavors and learning new recipes. Lastly, for pediatric patients requiring nutritional intervention, telemedicine could play a key role in offering innovative access to healthcare by providing necessary and strict follow-ups in order to optimize adherence to the suggested dietary patterns and increase the likelihood of successful treatment. Our proposed actions to spread the MD are schematized in Figure 4.

To better evaluate the role of the MD on inflammation, high-quality intervention studies are needed across different populations, particularly in children and adolescents with obesity and related comorbidities.

## Figures and Tables

**Table 2:** Literature evidence on the Mediterranean diet’s effects and adherence in pediatric populations with obesity.

Studies Evaluated	Study Design	Sample/Studies	Intervention and/or Study Design	MD Effects/Adherence in Pediatric Population
Velázquez-López et al. [119]	Open-label study	49 children and adolescents	24 children and adolescents were given the Mediterranean-style diet, while 25 were given the standard diet. Dietary calculations for children aged 3–10 and adolescents aged 10–18 were conducted using the Schofield equation.	Decrease in BMI, fat mass, blood glucose, total cholesterol, triglyceride, HDL- and LDL-cholesterol levels by following MD.
Kanellopoulou et al. [123]	Cross-section, population-based, observational study	1728 primary-school students (46% males, aged 10–12).	Family structure, dietary habits, and lifestyle were evaluated using questionnaires. MD adherence was determined using the KIDMED score. Children’s BMI was assessed according to the International Obesity Task Force classification.	Higher MD adherence acts as a protective factor against childhood overweight/obesity, particularly among children living with their families.
Notario-Barandiaran et al. [117]	Cross-sectional study	1801 and 1527 children who attended follow-up visits at age 4 and 8 years, respectively,	Dietary habits were evaluated at the age of 4 using a validated food frequency questionnaire. Adherence to MD was evaluated by rMED score. Children’s BMI was calculated according to the International Obesity Task Force classification.	High MD adherence at the age of 4 is linked to a reduced risk of overweight, obesity, and abdominal obesity by the age of 8.
Bacopoulou et al. [118]	Cross-sectional dietary intervention	1610 adolescents (12–17 years) in 23 public high schools	Nutritional education, promotion of physical activity, and raising awareness about body image for adolescent participants, their parents, schoolteachers, and health staff. Dietary assessment was evaluated using the KIDMED score, while BP, BMI, WC, and WHtR were measured at baseline and after a 6-month school-based intervention.	Decrease in overweight and obesity, mean systolic and diastolic BP, WC, and WHtR by following MD.
De Santi et al. [124]	Cross-sectional study	239 adolescent Italian students (119 boys and 120 girls, mean age: 12.1 ± 1.0)	Information on physical activity habits was gathered through a questionnaire. Adherence to the MD was assessed using the KIDMED score. Children’s BMI was determined based on the Cacciari classification.	Very low adherence to the MD among adolescents living in Mediterranean countries. association between MedDiet adherence, healthy behavior and normal weight status.
Seral-Cortes et al. [125]	Systematic-review	PubMed database was searched and only 1 study was included	Evaluated gene–MD interaction effects and its relationship with changes in body composition and metabolic parameters.	High adherence to the MD in individuals with a limited number of risk alleles was associated with a lower risk of adiposity and MetS.
López-Gil et al. [109]	Systematic review with meta-analysis	Four databases (PubMed, Scopus, Web of Science, and Cochrane Database of Systematic Reviews), and 15 studies were included	Assess the impact of Mediterranean diet-based interventions on anthropometric measurements and obesity indicators in children and adolescents.	Compared to the control group, the MD-based interventions showed small and significant reductions in BMI and significant reduction in the percentage of obesity MD-based interventions have a significant effect on reducing BMI.
Iaccarino et al. [127]	Systematic review	Several databases were systematically searched (PubMed, Scopus, Clinical Trials Results, Google Scholar and British Library Inside) and 58 studies published in the last 20 years were included	Compare MD adherence in children and adolescents with demographic and anthropometric variables (body composition, lifestyle, and diet adequacy).	10 of 26 papers reported that higher adherence to the MD was associated with lower BMI values or prevalence of overweight.
Lassale et al. [128]	Systematic review	Medline database was searched and 55 article were included	Impact of MD adherence on adiposity markers and obesity in children and adolescents.	More than 50% of studies found no significant association between MD adherence and adiposity.
Yurtdaş et al. [129]	Single-blind, randomized, two-arm, parallel dietary intervention	96 adolescents diagnosed with NAFLD (aged 11–18 years) were randomized to follow MD or conventional LFD (control diet) for 12 weeks.	Dietary status, anthropometric measurements, body composition, and biochemical parameters were assessed. Hepatic steatosis was diagnosed using ultrasonography.	Both MD and LFD decreased BMI, fat mass, hepatic steatosis, and insulin resistance, improved elevated transaminase levels, and had beneficial effects on inflammation and oxidative stress.

Abbreviations: BMI (body mass index); BP (blood pressure); WC (waist circumference); WHtR (waist-to-height ratio); MD (Mediterranean diet) MetS (metabolic syndrome), KIDMED (Mediterranean Diet Quality Index in children and adolescents), rMED score (relative Mediterranean diet score), NAFLD (non-alcoholic fatty liver disease), LFD (low-fat diet).
